# Beneficial Effects of Vitamin E Supplementation on Endothelial Dysfunction, Inflammation, and Oxidative Stress Biomarkers in Patients Receiving Hemodialysis: A Systematic Review and Meta-Analysis of Randomized Controlled Trials

**DOI:** 10.3390/ijms222111923

**Published:** 2021-11-03

**Authors:** Thi Thuy Uyen Nguyen, Ji-hyun Yeom, Won Kim

**Affiliations:** 1 Department of Histology, Embryology, Pathology and Forensic Medicine, Hue University of Medicine and Pharmacy, Hue University, Hue City 530000, Vietnam; nttuyen@hueuni.edu.vn; 2 Department of Internal Medicine, Jeonbuk National University Medical School, Jeonju 54896, Korea; mercifuldoc@gmail.com; 3 Research Institute of Clinical Medicine of Jeonbuk National University-Biomedical Research Institute of Jeonbuk National University Hospital, Jeonju 54907, Korea

**Keywords:** vitamin E, endothelial dysfunction, inflammation, oxidative stress, hemodialysis

## Abstract

Inflammation and oxidative stress are closely related to cardiovascular complications and atherosclerosis, and have the potential to lead to an increase in death in patients receiving hemodialysis. Vitamin E has antioxidant and anti-inflammatory properties. We conducted a systematic review and meta-analysis to assess the effects of vitamin E supplementation on endothelial dysfunction, inflammation, and oxidative stress biomarkers in adult patients receiving hemodialysis. We searched the MEDLINE, EMBASE, Web of Science, and Cochrane Library databases and identified randomized controlled trials of adult patients receiving hemodialysis until 30 August 2021. A total of 11 trials with 491 randomized patients were included. The pooled data indicated that vitamin E supplementation significantly decreased intercellular adhesion molecule-1 [standardized mean difference (SMD): −1.35; 95% confidence interval (CI): −2.57, −0.13; *p* = 0.03, I^2^ = 89%], vascular cell adhesion molecule-1 (SMD: −1.08; 95% CI: −2.05, −0.11; *p* = 0.03, I^2^ = 81%), C-reactive protein (SMD: −0.41; 95% CI: −0.75, −0.07; *p* = 0.02, I^2^ = 64%), and malondialdehyde (SMD: −0.76; 95% CI: −1.26, −0.25; *p* = 0.003, I^2^ = 77%) levels, but not interleukin-6 levels compared to those in the control group. Our results suggest that vitamin E supplementation may help alleviate oxidative stress and both vascular and systemic inflammation in patients receiving hemodialysis.

## 1. Introduction

Chronic inflammation and oxidative stress are strongly associated with the progression of chronic kidney disease and are common risk factors in patients with end-stage renal disease (ESRD) [1]. This may induce advanced cardiovascular complications and atherosclerosis through multiple pathogenic mechanisms [2,3]. Evaluation of this state through the levels of pro-inflammatory cytokines [interleukin-6 (IL-6), tumor necrosis factor alpha (TNF-α)], and acute-phase proteins [C-reactive protein (CRP)] may help predict the risk of all-cause mortality and cardiovascular mortality in patients with chronic renal failure receiving hemodialysis [4,5].

The levels of biomarkers of endothelial dysfunction and atherosclerosis, such as intercellular adhesion molecule-1 (ICAM-1) and vascular cell adhesion molecule-1 (VCAM-1), are increased in the serum of patients receiving hemodialysis [6]. Endothelial dysfunction increases vascular permeability and decreases nitric oxide bioavailability, leading to increased inflammation and thrombosis. Inflammation is also closely correlated with endothelial dysfunction and oxidative stress, which may contribute to increased cardiovascular morbidity and mortality in these patients [3,7,8]. In relation to the connection between inflammation and oxidative stress, anti-oxidative therapy may be a promising strategy to ameliorate the risks of cardiovascular disease in patients receiving maintenance hemodialysis [9]. Decreased kidney function is associated with an increased risk of thrombosis. Thrombosis is usually associated with venous thromboembolism, stroke, and coronary artery disease in patients with chronic kidney disease (CKD). Anti-thrombotic therapy may have a beneficial effect on thrombotic diseases in patients with CKD.

Vitamin E is a lipid-soluble antioxidant composed of eight different forms, including four tocopherols and four tocotrienols with high anti-inflammatory properties [10]. The pooled results of Asbaghi et al.’s study indicated the beneficial effect of vitamin E administration on the decrease in serum CRP concentration in adults [11]. In 2014, a meta-analysis indicated that vitamin E-coated dialyzer can help decrease inflammation and oxidative stress, reflected by reductions in serum CRP, IL-6, and thiobarbituric acid reactive substance levels in patients receiving hemodialysis [12]. Bergin et al. evaluated the pre- and post-treatment effects of vitamin E on malondialdehyde (MDA) levels [13]. They indicated that MDA levels are remarkably lower in patients receiving hemodialysis after receiving vitamin E supplementation. However, their pooled results did not show a comparison between the vitamin E treatment and control groups [13].

Recently, a randomized controlled clinical trial (RCT) demonstrated that vitamin E supplementation notably reduced biomarkers of vascular and systemic inflammation (ICAM-1, VCAM-1, CRP, IL-6, and TNF-α) [14]. In addition, Pirhadi-Tavandashti et al. reported significant reductions in serum ICAM-1 and VCAM-1 levels when comparing vitamin E consumption and placebos in patients receiving hemodialysis. However, there were no remarkable changes in CRP and IL-6 levels [15].

To the best of our knowledge, no meta-analysis has assessed the effects of vitamin E supplementation on biomarkers of endothelial dysfunction, and there remains inconsistency between clinical trials investigating the benefits of vitamin E treatment on inflammation and oxidative stress. Thus, we conducted a systematic review and meta-analysis of RCTs to evaluate the beneficial effects of vitamin E supplementation on biomarkers of endothelial dysfunction, inflammation, and oxidative stress in patients receiving hemodialysis.

## 2. Materials and Methods

### 2.1. Protocol and Registration

We registered in the International Prospective Register of Systematic Review database (Registration No. CRD42021262773) and conducted this systematic review and meta-analysis according to the Preferred Reporting Items for Systematic Reviews and Meta-Analysis (PRISMA) statement [16].

### 2.2. Eligibility Criteria

The ‘Population, Intervention, Comparison, Outcomes and Study’ framework was used to select studies that were eligible for inclusion in this systematic review. (1) Participants: adult patients with ESRD receiving hemodialysis; (2) Intervention: vitamin E supplementation; (3) Comparison: control group receiving placebo or not receiving study-related intervention; (4) Outcome: the primary outcome measures were biomarkers of endothelial dysfunction (ICAM-1 and VCAM-1), and the secondary outcomes included biomarkers of inflammation (CRP, IL-6) and oxidative stress (MDA); and (5) Study design: parallel groups and RCTs. All articles were published in English, without restrictions on the publication year. Studies were excluded if the outcome-of-interest was not assessed, or if they did not satisfy the eligibility criteria.

### 2.3. Information Sources and Search Strategy

We conducted a comprehensive literature search of four databases (MEDLINE, EMBASE, Web of Science, and Cochrane Library) and identified studies until 30 August 2021. The following text and medical subject heading terminologies were used to search for relevant articles: “vitamin E,” “tocopherol,” “tocotrienol,” and “hemodialysis”. For enhanced readability, the full search strategy is detailed in Appendix A. The duplicate results were cross-checked and excluded using Endnote X9 (Clarivates Analytics, Philadelphia, PA, USA). A secondary search was conducted using references from the relevant studies. Two investigators (T.T.U.N. and J.-h.Y.) performed and evaluated the search strategy.

### 2.4. Study Selection and Data Collection

According to the predetermined eligibility criteria, we used the PRISMA flow diagram to summarize the study collection processes. Two independent investigators reviewed the full-text articles after excluding unrelated titles and abstracts (T.T.U.N. and J.-h.Y.), and any disagreements were discussed and resolved to reach a consensus with the third author (W.K.). We used Microsoft Office Excel 2010 to extract the data and collect numerical data from the included studies. Two authors collected and examined the data. The divergent decisions were resolved by discussion and consensus with the third author (W.K.).

### 2.5. Data Items

The data from the included studies were independently collected according to the study source (authors, year of publication, and country), characteristics of the study and population (study design, sample size, proportion of men and women, mean age, mean body mass index, and hemodialysis duration), groups of trials (number of patients in each group, dosage, and duration of intervention), and outcomes. This process was conducted by two authors (T.T.U.N. and J.-h.Y.).

### 2.6. Risk-of-Bias Assessment

The Cochrane risk-of-bias tool for randomized trials version 2 (RoB2) was used to assess the quality of the included RCTs [17]. This tool was structured into five domains with the risk-of-bias decisions including “Low risk of bias”, “high risk of bias”, or “some concerns”. Two authors (T.T. and J.-h.Y.) independently conducted the methodological quality assessments. The disagreements were discussed by the three authors to arrive at a conclusion (T.T.U.N., J.-h.Y. and W.K.).

### 2.7. Data Analysis

All extracted data are shown as means ± standard deviations (SDs). If the data of the outcome-of-interest were provided as means with 95% confidence intervals (CIs) or medians and interquartile ranges, we converted them to means and SDs according to the formula of the Cochrane Handbook, chapter 6.5.2, or Wan et al.’s report, respectively [18,19]. Statistical analyses were conducted using Review Manager (RevMan) (Computer program) (Version 5.4, The Cochrane Collaboration (https://training.cochrane.org/online-learning/core-softwarecochrane-reviews/revman/revman-non-cochrane-reviews (accessed on 30 August 2021)), 2020). Standardized mean differences (SMDs) were used to evaluate the overall effect size of continuous data with different measurements or units. We extracted the means and SDs for changes between baseline and post-intervention. If these data were not provided, we estimated the means and SDs for changes following the recommendation of the Cochrane Handbook, chapter 6.5.2.8 [18].

The I square statistic (I^2^) was calculated to examine heterogeneity across studies; I^2^ ≤ 40% and *p*-value ≥ 0.1 were defined as low. In other cases, heterogeneity was considered moderate (40% < I^2^ ≤ 70%) or high (I^2^ > 70%) [20].

A random-effects model was used to estimate the pooled effect size for all the meta-analyses. Publication bias was assessed by Egger’s regression test (a formal statistical test of funnel plot asymmetry) using Comprehensive Meta-Analysis version 2.0 software [21]. Except for the heterogeneity test, *p* < 0.05 was considered significant for all statistical analyses in this study.

## 3. Results

### 3.1. Study Selection

**Figure 1:**
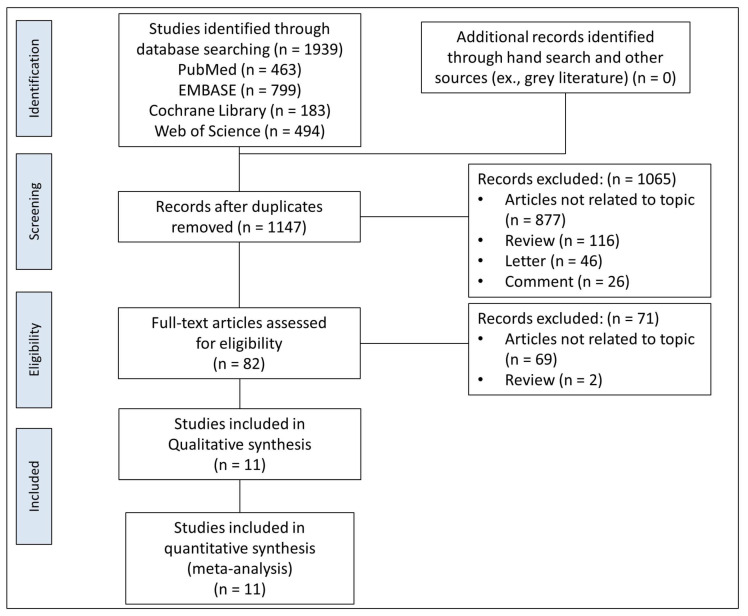
PRISMA flow diagram for selection of relevant trials.

The initial search results of the 4 electronic databases consisted of 1939 articles. After removing 792 duplicates, we screened the titles and abstracts and obtained 82 full-text articles for review. Of these, 71 articles that did not match the inclusion criteria were excluded, and 11 eligible studies were selected for the systematic review and meta-analysis. The PRISMA flowchart is shown in Figure 1.

### 3.2. Study Characteristics

The characteristics of the included studies are presented in Table 1. Eleven RCTs were published in English between 2006 and 2021, six of which were performed in Iran, with one each in the Czech Republic, Turkey, Spain, Philippines, and the USA. A total of 491 patients receiving hemodialysis were randomized into vitamin E (n = 246) or control (n = 245) groups, with the mean age ranging from 33 to 79 years. The duration of the intervention ranged from 2 to 20 weeks. The most commonly used dosage of vitamin E supplementation in the included trials was 400 IU daily. Among the included RCTs, 4 reported the effect of vitamin E supplementation on the levels of ICAM-1, 3 articles on VCAM-1, 9 articles on CRP, 5 articles on IL-6, and 6 articles on MDA, the data of which were pooled for meta-analysis. Other biomarkers such as E-selectin, P-selectin, pregnancy-associated plasma protein-A, monocyte chemoattractant protein-1 (MCP-1), superoxide dismutase (SOD), glutathione peroxidase, glutathione, and total antioxidant capacity (TAC) evaluated in several studies are listed in Table 1.

### 3.3. Risk-of-Bias Assessment

**Figure 2:**
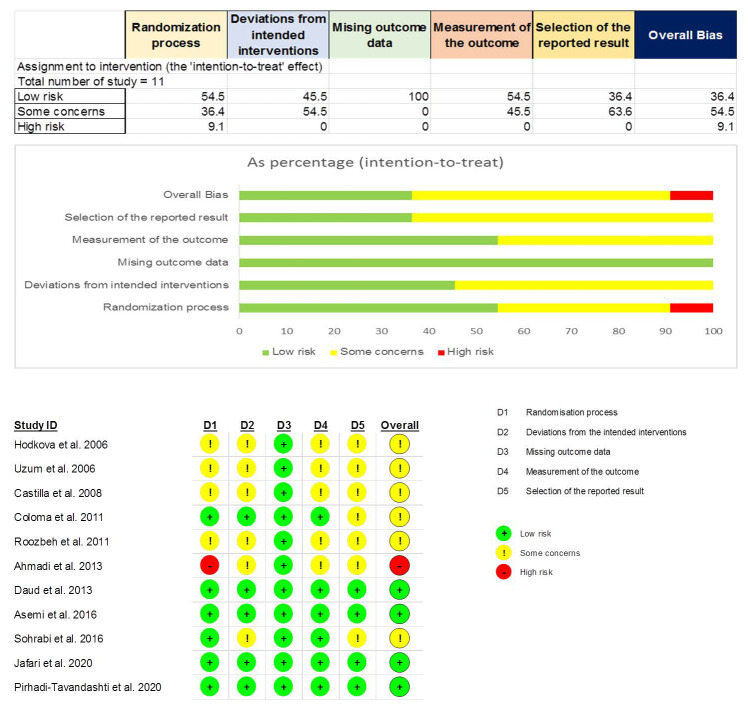
Risk of bias assessment by Cochrane risk-of-bias tool version 2 for included RCTs.

We summarized the quality assessment of the 11 RCTs included in Figure 2. Among them, four trials were judged as having a “low risk of bias” (36.4%), six trials as having “some concerns” (54.5%), and one trial as having a “high risk of bias” (9.1%). Notably, all trials were assessed as “low risk” in the domain of missing outcome data, and only the domain of the randomization process received a “high risk” assessment from one trial.

### 3.4. Meta-Analysis

#### 3.4.1. Effect of Vitamin E Supplementation on Biomarkers of Endothelial Dysfunction

**Figure 3:**
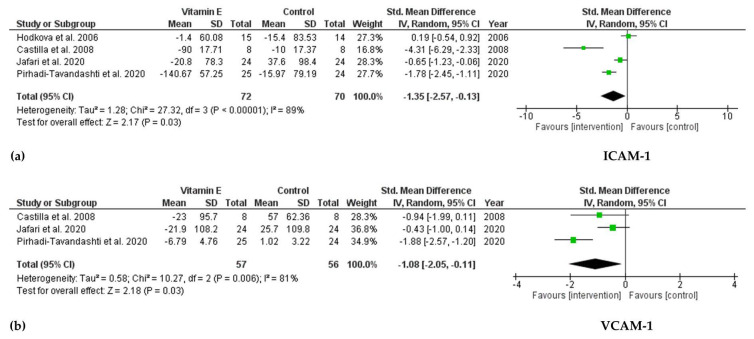
Forest plot for effect of vitamin E supplementation on (**a**) ICAM-1 and (**b**) VCAM-1 in hemodialysis patients.

Four RCTs investigated ICAM-1 levels. The pooled results indicated a significant reduction in serum ICAM-1 levels after taking vitamin E (SMD = −1.35; 95% CI: −2.57, −0.13; *p* = 0.03, I^2^ = 89%). In a meta-analysis of three trials investigating VCAM-1, there was a remarkable decrease in serum VCAM-1 levels in the vitamin E group compared with that in the control group (SMD = −1.08; 95% CI: −2.05, −0.11; *p* = 0.03, I^2^ = 81%) (Figure 3). No publication bias was observed based on Egger’s test for ICAM-1 and VCAM-1 (*p* = 0.372 and *p* = 0.828, respectively).

#### 3.4.2. Effect of Vitamin E Supplementation on Biomarkers of Inflammation

**Figure 4:**
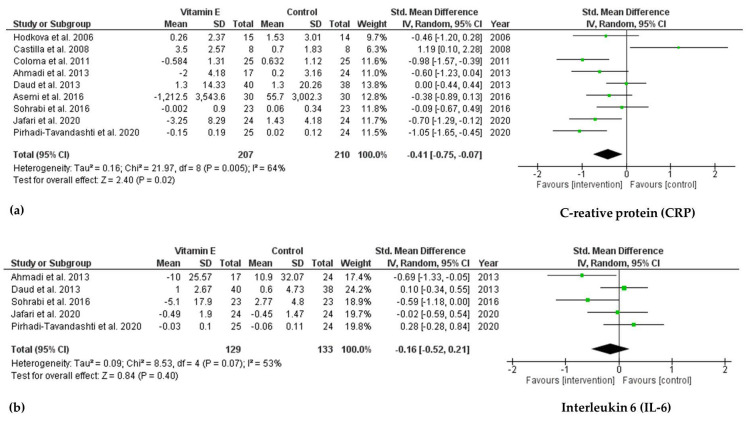
Forest plot for effect of vitamin E supplementation on biomarkers of inflammation (**a**) Creative protein (CRP) and (**b**) Interleukin 6 (IL-6) in hemodialysis patients.

As shown in Figure 4, a significant decrease in CRP levels was found in the vitamin E intervention group compared to that in the control group (SMD = −0.41; 95% CI: −0.75, −0.07; *p* = 0.02, I^2^ = 64%). However, there was no significant change after vitamin E supplementation in IL-6 levels (SMD = −0.16; 95% CI: −0.52, 0.21; *p* = 0.40, I^2^ = 53%). No publication bias was observed based on Egger’s test for CRP and IL-6 (*p* = 0.579 and *p* = 0.229, respectively).

#### 3.4.3. Effect of Vitamin E Supplementation on Biomarkers of Oxidative Stress

**Figure 5:**
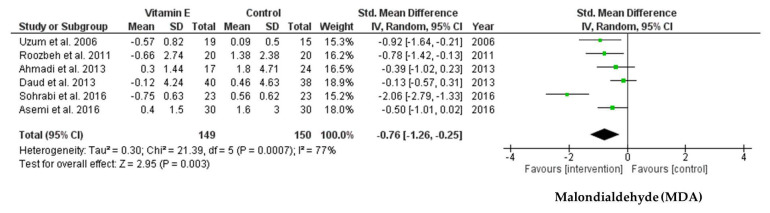
Forest plot for effect of vitamin E supplementation on malondialdehyde (MDA) in hemodialysis patients.

The meta-analysis of six RCTs revealed a significant difference in MDA levels following vitamin E supplementation when compared between the intervention and control groups (SMD = −0.76; 95% CI: −1.26, −0.25; *p* = 0.003, I^2^ = 77%) (Figure 5). Egger’s test for this meta-analysis suggested no publication bias (*p* = 0.059).

## 4. Discussion

This review pooled the data of 11 RCTs in patients receiving hemodialysis to determine the beneficial effect of vitamin E supplementation on the reduction in biomarkers of inflammation and oxidative stress. Our study is the first meta-analysis to demonstrate the effect of this supplementation on significantly reducing biomarkers of vascular inflammation (ICAM-1 and VCAM-1) in patients receiving hemodialysis. In addition, by evaluating CRP and MDA levels, we also showed remarkable evidence to assess the benefits of vitamin E in decreasing systemic inflammation and oxidative stress.

Hemodialysis may improve survival in patients with ESRD, but increased inflammation and oxidative stress are associated with several complications, such as atherosclerosis and malnutrition, and contribute to their acceleration in these patients [14,31,32]. Therefore, the use of adjuvant therapy in patients receiving hemodialysis to reduce inflammation and oxidative stress is a matter of concern. Vitamin E is a widely studied antioxidant. Many clinical trials have used vitamin E to coat dialysis membranes, the effectiveness of which on inflammation and oxidative stress has been demonstrated by the pooled results of previous meta-analyses [12,33,34,35]. In addition, several trials have investigated the effects of the oral administration of vitamin E in patients receiving hemodialysis. Based on the RCTs, we show the most valuable evidence of the benefits of vitamin E supplementation through a meta-analysis.

Adhesion molecules, including ICAM-1 and VCAM-1, which may indicate endothelial dysfunction, are increased under conditions of high inflammation and oxidative stress. Patients receiving hemodialysis are more likely to have higher atherosclerotic cardiovascular disease prevalence [14,15,36]. The serum concentrations of vascular inflammatory markers have been evaluated in patients with ERSD [37]. Their high levels are related to an increase in cardiovascular-induced mortality [38,39]. Our findings demonstrated a significant reduction in serum ICAM-1 and VCAM-1 levels in patients receiving hemodialysis and vitamin E supplementation compared with that in the group without intervention. The clearance of oxidative stress via the antioxidant capacity of vitamin E may reduce the gene expression of these molecules [15,40,41]. 

Elevated levels of acute-phase proteins or pro-inflammatory cytokines, such as CRP, IL-6 are evidence of systemic inflammation in patients receiving dialysis. An increase in this inflammatory factor during hemodialysis sessions is linked to high mortality [42]. In this review, we found a reduction in CRP levels after vitamin E supplementation. This is in line with the results of a previous meta-analysis by Asbaghi et al. of adults in general and patients receiving hemodialysis [11]. However, there was a slight methodological difference in their study compared to the present study. We aggregated the data to compare the effects between patients who received only vitamin E treatment and the control group, while they summarized data corresponding to both patients who received vitamin E and those who received vitamin E combined with another substance and compared them with the control group [11]. Similar to the previous study, we evaluated the change in IL-6 level. However, there was no evidence of a significant reduction in IL-6 levels in patients receiving vitamin E supplementation.

The imbalance between pro-oxidant and antioxidant systems in patients receiving hemodialysis leads to an increase in oxidative stress, which is associated with both pathophysiologic mechanism of ESRD and hemodialysis techniques [43]. MDA is the end-product of the reaction between reactive oxygen species and polyunsaturated fatty acids. This may interact with proteins and nucleic acids and is implicated in the pathogenesis of several disorders, including atherosclerosis [44]. Our meta-analysis indicated that vitamin E supplementation significantly reduces MDA levels in the intervention groups. Although a previous meta-analysis showed a decrease in MDA levels in the serum of patients receiving hemodialysis when comparing results between pre- and post-intervention, the comparison of effectiveness between vitamin E supplementation and control groups is unclear. To date, we pooled the results from RCTs to evaluate and demonstrate a significant difference in MDA levels between the vitamin E treatment and non-treatment groups.

Patients receiving hemodialysis are a population with many disorders related to inflammation, oxidative stress, and malnutrition. Inflammatory status can induce reactive oxygen species (ROS) and lead to increased consumption of antioxidants. On the other hand, oxidative stress can influence inflammation through the production of pro-inflammatory cytokines and aggravate other syndromes, such as atherosclerosis and malnutrition. Vitamin E is one of many nutrients that is restricted or disrupted in metabolism, intake, and clearance in hemodialysis patients [45]. Moreover, gastrointestinal problems in this population may cause poor absorption, which leads to low plasma vitamin E levels in certain patients [46,47]. Vitamin E is a lipid-soluble antioxidant with anti-inflammatory properties. It can act as an ROS scavenger and may control lipid peroxidation and oxidative stress. Therefore, vitamin E supplementation may improve vitamin E deficiency due to malnutrition and provide antioxidant and anti-inflammatory effects in patients receiving hemodialysis.

An increased risk of thromboembolism and stroke occurs when renal function is decreased. CKD patients, especially those on renal replacement therapy, exhibit an increased risk of thrombosis, including myocardial infarction, stroke, deep vein thrombosis, and pulmonary embolism. These complications may be due to the high incidence of atrial fibrillation, hypercoagulability or unknown factors in these patients. Supplementation with vitamin E may suppress thromboembolic events. The incidence of venous thromboembolism was decreased in women treated with vitamin E [48]. Vitamin E was associated with inhibition of platelet aggregation and thrombin formation [49,50]. Therefore, vitamin E supplementation may have a beneficial effect on thrombotic events in patients with CKD.

Despite these remarkable results, our study has some limitations. In this systematic review, we included several studies that evaluated antioxidant defenses using antioxidant markers (GSH, SOD, and TAC), and inflammatory biomarkers such as TNFα and MCP-1. However, because of the small number of RCTs, we were unable to perform meta-analyses to assess the effect of vitamin E supplementation on these markers. Additionally, the number of RCTs and sample sizes included in the pooled analyses remain limited. Therefore, further studies with larger sample sizes are required.

The elevated plasma IL-6 level is commonly observed in CKD patients. Increased serum IL-6 level is largely caused by many variables such as the increased production resulting from oxidative stress, chronic inflammation, and the decreased renal clearance of IL-6 in CKD [51]. Vitamin E supplementation in diabetic patients suppresses the serum level of IL-6 by regulation of oxidation and inflammation [52]. However, the present meta-analysis study showed that there are no significant differences in serum IL-6 levels between the untreated and patients treated with vitamin E supplementation. This could be linked to several factors, including the study duration, the number of enrolled patients, dosage of vitamin E used, and the baseline levels of the inflammatory state. In addition, residual renal function may be an additional factor influencing the outcome [53]. We believe that further study is needed to measure the effect of vitamin E in CKD.

The heterogeneity across the included studies was moderate and high. The type, dosage, and duration of vitamin E supplementation differed among the studies. We were unable to provide evidence and recommend an appropriate dosage and duration for clinicians. Further studies should be conducted to compare the effectiveness of different types, doses, and durations of vitamin E supplementation. Although the percentage of studies with a high risk of bias was 9.1%, 54.5% of the included studies had “some concerns”. Based on the Cochrane risk-of-bias assessment, we have some recommendations to improve the quality of RCTs, such as using random methods for generating the allocation sequence and blinding of the outcome assessors and participants, following the pre-specified analysis plan before unblinding the data.

## 5. Conclusions

In the current meta-analysis, our results demonstrated that vitamin E supplementation significantly decreases the levels of biomarkers of endothelial dysfunction and vascular inflammation (ICAM-1 and VCAM-1), systemic inflammation (CRP), and oxidative stress (MDA) compared to those in the control group. However, it does not influence IL-6 levels. Further RCTs of higher quality are needed to confirm these findings.

## Figures and Tables

**Table 1:** Characteristics of the included randomized controlled trials in the systematic review.

Author, Year	Country	Type of Study	Population (M/F)	Mean Age (Years)	Mean BMI (kg/m^2^)	Hemodialysis	Intervention Group (n)	Control Group (n)	Outcome	Duration (Weeks)
Hodkova et al. 2006 [22]	Czech Republic	randomized controlled trial	29 (10/19)	62	NA	HD thrice weekly, 4 h/session, Kt/V >1.2	alpha-tocopherol 400 mg = 888 IU of vitamin E daily (n = 15)	No intervention (n = 14)	CRP, ICAM-1, E-selectin, PAPP-A	5
Uzum et al. 2006 [23]	Turkey	prospective, randomized placebo-controlled trial	34 (18/16)	46	23	HD thrice weekly, 4 h/day	300 mg vitamin E daily (n = 19)	Placebo (n = 15)	MDA, SOD	20
Castilla et al. 2008 [24]	Spain	randomized controlled trial	32 (16/16)	range (33–79)		HD thrice weekly for 3.5–4.5 h/session	800 IU/day α-tocopherol (n = 8)	No intervention (n = 8)	CRP, ICAM-1, VCAM-1, MCP-1	2
Coloma et al. 2011 [25]	Philippines	Prospective randomized double-blind placebo-controlled clinical trial	50 (36/14)	60	22	HD 4 h/session, 2 sessions/week, Kt/V: 1.61 ± 0.4066 (intervention); 1.7 ± 0.4594 (placebo)	vitamin E (400 IU) daily (n = 25)	Placebo (n = 25)	CRP	8
Roozbeh et al. 2011 [26]	Iran	randomized controlled trial	40 (27/13)	44	NA	HD thrice weekly	vitamin E 400 IU daily (n = 20)	No intervention (n = 20)	MDA, GPX	3
Ahmadi et al. 2013 [27]	Iran	randomized placebo-controlled trial	41 (20/21)	47	25.5	at least 2 times weekly for at least 1 year	vitamin E (400 IU) daily (n = 17)	Placebo (n = 24)	CRP, IL-6, MDA	8
Daud et al. 2013 [28]	USA	randomized, double-blind, place bo-controlled, parallel trial	81 (43/38)	58.5	29.5	Kt/V: 1.45 ± 0.20 (intervention); 1.48 ± 0.26 (placebo)	vitamin E tocotrienol-rich fraction (TRF) (180 mg tocotrienols, 40 mg tocopherols) daily (n = 40)	Placebo (n = 38)	CRP, IL-6, MDA	16
Asemi et al. 2016 [29]	Iran	randomized double-blind placebo-controlled clinical trial	60 (40/20)	60	NA	NA	400 IU/day alpha-tocopherol (n = 30)	Placebo (n = 30)	CRP, MDA, TAC, GSH	12
Sohrabi et al. 2016 [30]	Iran	randomized, controlled, nonblinded, parallel trial	46 (23/23)	57	22.5	NA	vitamin E (600 IU) 3 times a week (n = 23)	No intervention (n = 23)	CRP, IL-6, MDA	8
Jafari et al. 2020 [14]	Iran	randomized, double-blind, placebo-controlled clinical trial	48 (23/25)	54	23	HD thrice weekly Kt/V: 1.41 ± 0.71 (intervention); 1.60 ± 0.10 (placebo)	vitamin E soft gel (400 IU) daily (n = 24)	Placebo (n = 24)	CRP, IL-6, TNF-α, ICAM-1, VCAM-1, P-selectin	8
Pirhadi-Tavandashti et al. 2020 [15]	Iran	randomized, double-blinded, and placebo-controlled clinical trial	49 (15/34)	45	26	NA	600 IU alpha-tocopherol soft gel (n = 25)	Placebo (n = 24)	CRP, ICAM-1, VCAM-1, IL-6	10

HD, hemodialysis; CRP, C-reactive protein; ICAM-1, intercellular adhesion molecule-1; PAPP-A, pregnancy-associated plasma protein-A; MDA, malondialdehyde; SOD, superoxide dismutase; VCAM-1, vascular cell adhesion molecule 1; MCP-1, monocyte chemoattractant protein-1; GPX, glutathione peroxidase; IL-6, interleukin 6; NA, not available; TAC, total antioxidant capacity; GSH, glutathione; TNF-α, tumor necrosis factor alpha.

## Data Availability

All data are reported in this manuscript.

## Appendix A

**Table A1:** Detailed search strategies for each database. MeSH terms, search terms, and combinations of the two were used for each database search.

Database	Detailed Search Strategies	Records Founded
MEDLINE/PUBMED	(“vitamin e”[MeSH Terms] OR vitamin E[Text Word] OR “tocopherols”[MeSH Terms] OR tocopherol[Text Word] OR “tocotrienols”[MeSH Terms] OR tocotrienol[Text Word]) AND (“renal dialysis”[MeSH Terms] OR hemodialysis[Text Word])	463
EMBASE	(“vitamin E” OR “tocopherol” OR “tocotrienol”) AND hemodialysis	799
Cochrane Library	(“vitamin E” OR “tocopherol” OR “tocotrienol”) AND hemodialysis	183
Web of Science	(“vitamin E” OR “tocopherol” OR “tocotrienol”) AND hemodialysis	494

Ultimately, 1939 records were found, 463 from MEDLINE/PubMed, 799 from EMBASE, 183 from Cochrane Library, and 494 from the Web of Science. Studies were further selected according to the inclusion criteria listed in the Material and Methods (Figure 1).
